# Nanocomposite hydrogel microneedles: a theranostic toolbox for personalized medicine

**DOI:** 10.1007/s13346-024-01533-w

**Published:** 2024-02-20

**Authors:** Catarina F. Martins, Clara García-Astrain, João Conde, Luis M. Liz-Marzán

**Affiliations:** 1 ToxOmics, NOVA Medical School, Faculdade de Ciências Médicas, NMSFCM, Universidade NOVA de Lisboa, Lisbon, Portugal; 2 CIC biomaGUNE, Basque Research and Technology Alliance (BRTA), 20014 Donostia-San Sebastián, Spain; 3 Centro de Investigación Biomédica en Red, Bioingeniería, Biomateriales y, Nanomedicina (CIBER-BBN), 20014 Donostia-San Sebastián, Spain; 4 Ikerbasque, Basque Foundation for Science, 48009 Bilbao, Spain

**Keywords:** Microneedles, Nanocomposite hydrogels, Theranostics, Cancer

## Abstract

Due to the severity and high prevalence of cancer, as well as its complex pathological condition, new strategies for cancer treatment and diagnostics are required. As such, it is important to design a toolbox that integrates multiple functions on a single smart platform. Theranostic hydrogels offer an innovative and personalized method to tackle cancer while also considering patient comfort, thereby facilitating future implementation and translation to the clinic. In terms of theranostic systems used in cancer therapy, nanoparticles are widely used as diagnostic and therapeutic tools. Nanoparticles can achieve systemic circulation, evade host defenses, and deliver drugs and signaling agents at the targeted site, to diagnose and treat the disease at a cellular and molecular level. In this context, hydrogel microneedles have a high potential for multifunctional operation in medical devices, while avoiding the complications associated with the systemic delivery of therapeutics. Compared with oral administration and subcutaneous injection, microneedles offer advantages such as better patient compliance, faster onset of action, and improved permeability and efficacy. In addition, they comprise highly biocompatible polymers with excellent degradability and tunable properties. Nanoparticles and microneedles thus offer the possibility to expand the theranostic potential through combined synergistic use of their respective features. We review herein recent advances concerning processing methods and material requirements within the realm of hydrogel microneedles as theranostic platforms, various approaches toward cancer therapy, and the incorporation of nanoparticles for added functionality.

**Graphical Abstract:**

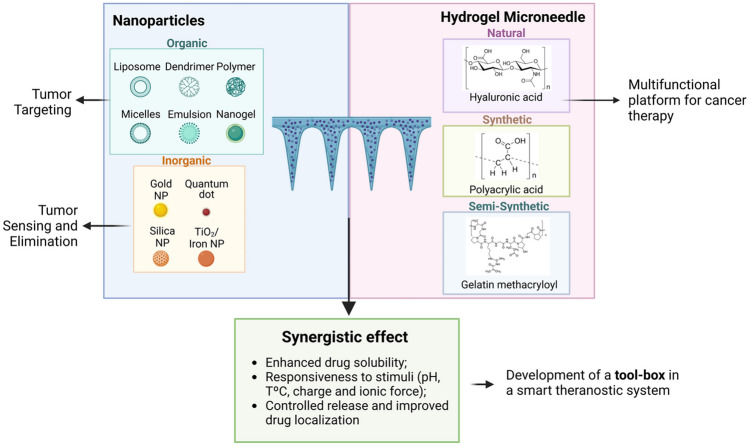

## Introduction

Broadly considered, the term cancer refers to a set of diseases in which malignant cells grow abnormally fast and spread throughout the body. Cancer is the second-leading cause of mortality worldwide. Among many different types, lung cancer is the most frequently diagnosed variety, followed by breast cancer, prostate cancer, and colorectal cancer [1]. Cancer is considered a heterogeneous disease due to its wide variability, triggered by various endogenous and exogenous factors. Such a heterogeneity leads to a diverse biological presentation, epidemiology, response to treatment, and prognosis [1–4]. Therefore, there is still a need for effective drug delivery strategies that ensure overall therapeutic efficacy. Among the different types of treatment administration, intravenous and oral are preferentially chosen, but even these routes pose issues in terms of drug biodistribution into the target site, associated with poor pharmacokinetics and clearance [5]. In this context, transdermal patches appear as a promising solution, offering a simple and efficient drug delivery strategy. The main concept is related to macroscale delivery systems that can be locally implanted on the tumor tissue, thereby avoiding the complications associated with the systemic delivery of therapeutics. More specifically, microneedles (MNs) are particularly attractive because they are minimally invasive, with better patient compliance and a faster onset of action, thus permitting enhanced drug delivery into the target site, while reducing medical costs [5].

MNs can be used to efficiently deliver both small molecules and macromolecules, such as chemotherapeutics, proteins, and genetic material, along with nanoparticle-based anticancer therapies. Compared with conventional administration and subcutaneous injection, MN patches offer several advantages, such as a controlled delivery rate, low drug concentration in blood, high permeability, reduced toxicity side effects, and avoidance of first-pass hepatic metabolism, with minimal pain and discomfort [6]. In terms of cancer therapy, MNs have been used for localized therapy, enabling controlled release and minimizing systemic side effects, for combination therapy based on its versatility for comprehensive treatment, and for photothermal therapy through the incorporation of nanomaterials allowing localized hyperthermia for selective destruction of cancer cells upon heating. Therefore, MNs open new avenues toward personalized medicine and targeted treatment, thereby improving the effectiveness and patient experience during cancer therapy. Notwithstanding, MNs also present certain limitations, such as the need to ensure biocompatibility to prevent adverse reactions and inflammation, optimization of the loading capacity to guarantee the supply of therapeutic agents for effective treatment, manufacturing scalability, following regulatory considerations to reach clinical translation while ensuring safety and efficacy, as well as improving patient acceptance [6, 7]. In terms of design, MNs require the use of a biocompatible matrix material to obtain a solid pointed structure with sufficient mechanical strength to penetrate the skin and create microchannels that facilitate drug diffusion. The design of the pointed structure should be tailored for different applications. Solid MNs have the advantage of a simpler fabrication and minimum invasiveness compared to traditional drug delivery methods. On the other hand, coated MNs comprise a solid base surrounded by a drug-loaded coating, which gets dissolved upon skin penetration, triggering the release of the therapeutic payload in a more controlled manner. Alternatively, hollow MNs feature an internal microchannel that allows the passage of liquid formulations, and as such their mechanism is based on directly injecting the drug into the desired site, allowing for a more precise delivery action. Finally, dissolving MNs are typically made of a water-soluble material that dissolves upon penetration in the skin, releasing the drug. Dissolving MNs have the advantage of being more patient-friendly because there is no need to remove them after treatment [6, 7].

Among the different types of materials suitable for MN fabrication, hydrogel-forming MNs (HFMs) have gained attention due to their hydrophilic nature, which ensures a sustained release mechanism. Moreover, the versatility in material composition, high loading capacity and ease of fabrication makes hydrogels ideal candidates for MN preparation [8, 9]. These HFMs swell and dissolve upon insertion into the skin, resulting in a more sustained drug release [9]. Hydrogels have a high degree of porosity, which can be tuned through crosslinking of the constituting polymers, which additionally provides mechanical strength, adhesive properties, stability, and protection of the therapeutic agent. With a highly tunable physical structure, the applications of hydrogels are almost unlimited, rendering them a useful toolbox for biomedical applications and MN fabrication [10, 11]. Regarding cancer research, HFMs are ideal substrates for the integration of diagnostics, therapy, and/or imaging into a single system, which is often referred to as a theranostic platform [12, 13]. Among other advantages, HFMs enable long-term diagnostic capabilities and continuous monitoring of cancer and pharmacokinetics, eventually improving the patient’s response to treatment. Additionally, HFMs may even avoid the need for surgery and biopsy collection, thereby reducing the cost and risk associated with invasive methods [14–18].

**Fig. 1:**
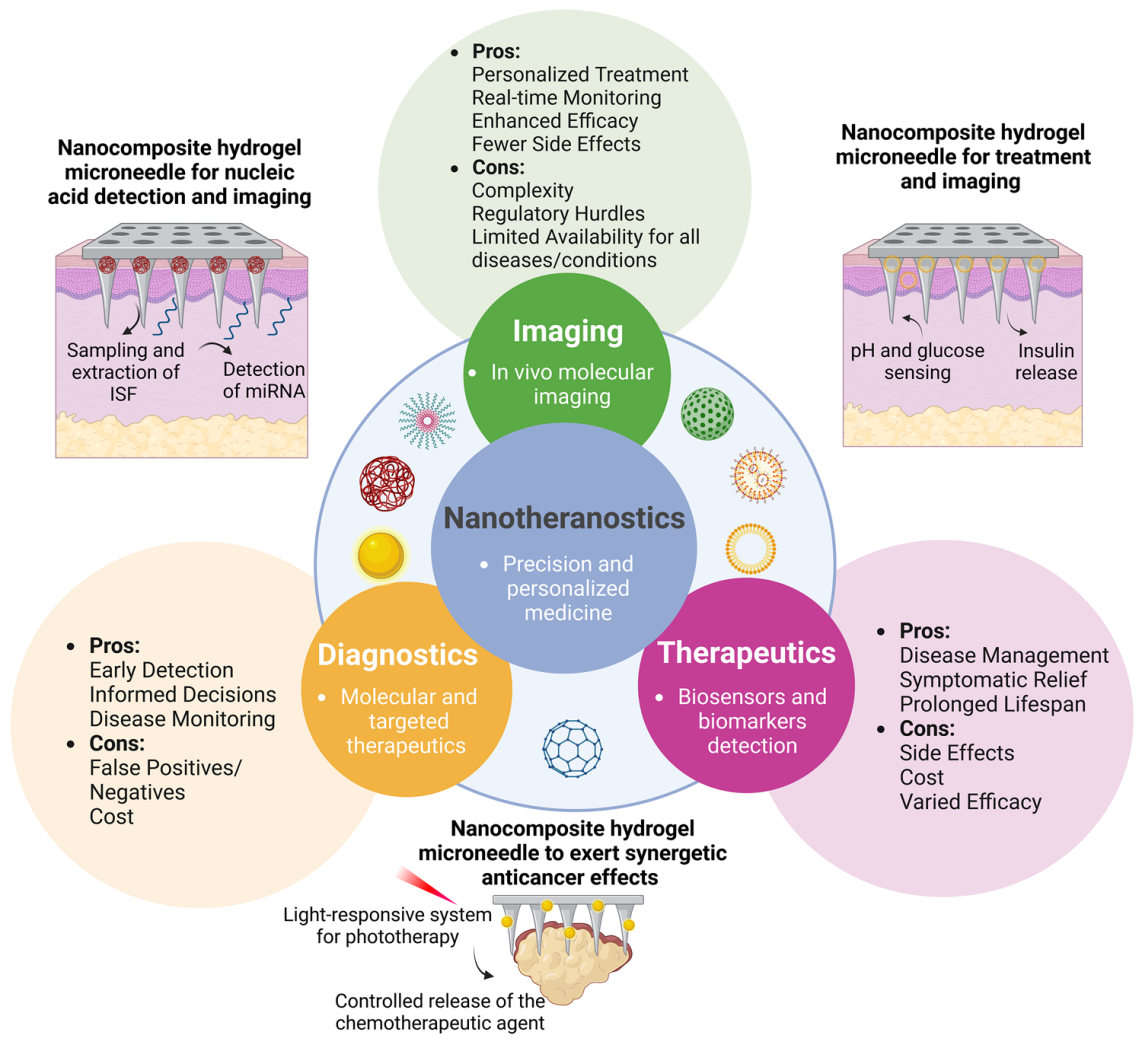
Schematic view of the term nanotheranostics, comparing the advantages (pros) and drawbacks (cons) of traditional therapies and diagnostic methods versus integrative nanotheranostic approaches in precision medicine, with the representation of hydrogel microneedle systems

To improve diagnosis, prevention, and disease treatment, HFMs can also be combined with nanomaterials [19, 20]. The term nanotheranostics refers to the combination of diagnosis, imaging, and therapeutic activity of nanomaterials, integrated simultaneously in a single system to combat a certain disease [21]. This approach not only aids in treatment planning but also enables real-time monitoring of therapeutic responses, thereby facilitating personalized treatments (Fig. 1) [21–23]. Within the rich nanotechnology toolbox for diagnosing and treating cancer, various multifunctional nanocarriers have emerged as versatile tools for a wide array of innovative therapeutic strategies in drug delivery [24]. The advantages of such nanocarriers encompass a high loading efficiency, facilitating combination therapy, ensuring controlled release and prolonged circulation in the body, as well as the ability to target specific sites [25, 26]. Therefore, it remains imperative to discuss the interplay between nanoparticles (NPs) and the host hydrogel, to consider the processing methods employed for nanocomposite HFMs fabrication, and to evaluate their collective impact on the overall performance of the system.

## Hydrogel selection and material requirements

The choice of a specific hydrogel for microneedle applications is a critical design aspect, because it influences the resulting mechanical properties, drug release kinetics, and biocompatibility [27]. The formulation of an ideal MN from hydrogels requires a number of functional features, including the following: a high absorption capacity in saline solutions, a tunable rate of absorption (preferred hydrogel pore size and porosity) depending on the application requirement, highest absorbency under load, lowest soluble content with high resistance to dissolution in water, low cost and ease of fabrication, highest durability and stability in the swelling environment and during storage, tailored biodegradability without the formation of toxic species, pH neutrality after swelling, and absence of color [28–30]. In fact, the ideal hydrogel for MN formulation should efficiently absorb physiological fluids to ensure that the MNs can swell upon application, thereby facilitating drug release or other functionalities [31]. Depending on the specific therapeutic needs, one might require faster or slower rates of absorption. Factors such as particle size and porosity should be adjustable to fit diverse therapeutic applications. Besides, HFMs should maintain their absorbency when subjected to external pressure, thus ensuring consistent performance irrespective of external conditions. By reducing the presence of soluble components in hydrogels, the potential leaching of unwanted substances during HFM application can be minimized, which is crucial for safety. Indeed, HFMs need to maintain their structure both in a swelling environment and during storage, to ensure their performance while avoiding premature degradation [32–36]. Regarding mechanical properties, the hydrogel should have sufficient rigidity to penetrate the skin or target tissue, but also suitable flexibility to accommodate dynamic movements.

Table 1 summarizes the general specifications to fabricate HFMs for drug delivery. The optimal values for these parameters will depend on the specific requirements of the HFM application, the type of drug to be delivered, and the desired release profile. Conducting in-depth studies and optimization based on these ranges can help tailor hydrogel formulations for effective and safe drug delivery through microneedles. Finally, the hydrogels must be biocompatible to minimize any adverse reactions or inflammation upon insertion into the tissue, which can be evaluated through in vitro assays such as enzymatic degradation tests, cell viability and cytotoxicity assays, as well as in vivo patch testing, cytokine expression analysis, histological analysis, and biocompatibility with animal models.

**Table 1:** Summary of the required properties and their respective ideal ranges for HFM fabrication

**Property**	**Ideal Range**	**References**
Needle length (µm)	500–800	[9, 37]
Tip diameter (µm)	60–160	[9, 37]
Insertion force (N)	0.08–3.04	[9, 37]
Gelation time (min)	1–30	[38]

In general, hydrogels can be classified in terms of their source, degree of crosslinking, composition, and charge. Numerous reviews have been published, establishing hydrogel classifications and reporting in detail their characteristics [39, 40]. We focus here on the most common hydrogels employed for HFM fabrication and the requirements to achieve the desired characteristics. When developing a hydrogel for HFM fabrication, the most relevant factors are the degree of crosslinking and the polymer source used to form the matrix. Regarding the polymer source, synthetic hydrogels such as poly(vinyl alcohol) (PVA), poly(vinyl) pyrrolidone (PVP), polyacrylic acid (PAA), and poly(N-isopropylacrylamide) (PNIPAAm) have been widely employed for HFM preparation. Synthetic hydrogel-forming polymers have tunable chemical properties but lack inherent fundamental biological cues, thus requiring their conjugation to cell-binding peptides such as arginylglycylaspartic acid (RGD). On the other hand, natural hydrogels such as hyaluronic acid or gelatin retain their native cell-binding sites but have low reproducibility and batch-to-batch variation. Lastly, semi-synthetic hydrogels, such as gelatin methacryloyl (GelMA) or methacrylated hyaluronic acid (HAMA), retain the biocompatibility and bioactive features of the source polymer, while ensuring the necessary mechanical stability and tunability due to rapid crosslinking [41–51]. Other natural polymers obtained from renewable resources, such as chitosan or cellulose derivatives, have been also reported for HFM fabrication because they are non-toxic and carry suitable functional groups for chemical modification [45]. Crosslinking is a crucial step in the fabrication of HFMs because it imparts mechanical strength and stability to the hydrogel structure. Hydrogel crosslinking also offers the possibility to control the release of therapeutics through fine-tuning the swelling degree of the material. Various crosslinking techniques can be employed to achieve the desired properties in HFMs, which are closely related to the polymer composition. Table 2 summarizes examples of crosslinking strategies for polymers used in HFMs, together with the benefits and drawbacks of each type of polymer [41–51].

**Table 2:** Summary of different polymers used for HFMs, their main properties and reported applications

**Polymer**	**Benefits**	**Drawbacks**	**Crosslinking**	**Application**	**Ref**
Gelatin	• Ease of fabrication • Highly biocompatible	• Limited drug load	Physical	• Transdermal drug delivery	[41]
Hyaluronic acid	• Highly biocompatible • Bioresponsive • Promotes skin hydration	• Bioinert • Rapid degradation • Costly	Physical	• Photothermal therapy • Drug delivery • Imaging	[42–44]
HAMA	• Improved degradation • Biocompatible	• Costly	Photo-crosslinking	• Biomarker sensing	[42]
GelMa	• Biocompatible • Sustained drug release • Tuneable mechanical properties	• Bioinert • Limited stability • Potential immunogenicity	Photo-crosslinking	• Transdermal drug delivery	[45]
Chitosan	• Biocompatible • Adhesive properties	• Limited mechanical properties	Chemical, enzymatic	• Transdermal drug delivery	[46]
PAA	• Good swelling capacity • Sustained drug release	• Potential skin irritation	Chemical	• Vaccine release	[47]
PNIPAAm	• Thermoresponsive, • Biocompatible • Chemically versatile	• Bioinert • Rapid release	Chemical	• Transdermal drug delivery	[48, 49]
PVA	• High swelling • Controlled release	• Limited biodegradability	Chemical	• Transdermal drug delivery • Photothermal therapy	[50]
PVP	• Biocompatible • Adhesive properties	• Limited mechanical properties • Limited biodegradability	Chemical	• Transdermal drug delivery • Immunotherapy	[51, 52]

## Hydrogel microneedle design and processing methods

When designing and developing a MN patch, it is important to consider geometrical features (length, diameter, tip size, and shape), whether it is a solid, hollow, side-opened, conical, or beveled tip, material composition, fabrication feasibility, application, layout of the arrays, MN density, total number of MN tips, and surface coating. The ultimate design will thus depend on the limitations imposed by the processing method, as well as the mechanical, physical, and chemical properties of the source material [8, 53]. The shape of MNs is an important aspect of MN design because it determines how much force can be applied before it breaks. The diameter and angle of the tip, as well as the height and base dimensions, determine the safe and reliable insertion of the MN into the skin. Generally, a smaller tip diameter, smaller tip angle, as well as a high ratio between the height and the base width, will result in successful needle insertion [54–56]. Most MNs to date have been fabricated with heights shorter than 1 mm, yet sufficient to access interstitial fluids (ISF), capillary blood, and to deliver therapeutic agents. When inserting MNs in the tissue to create microchannel arrays, it is important to consider some variables that may affect the flow, such as blood viscosity, contact angle, hydrodynamic diameter, and driving forces including surface tension. In addition, due to the elastic nature of tissues and their irregular surface, which varies for each individual, but also with age and body location, the efficient penetration of MNs to the desired depth without fracture and with high accuracy, may require an applicator to facilitate tissue penetration in a controlled and reproducible manner [56].

**Fig. 2:**
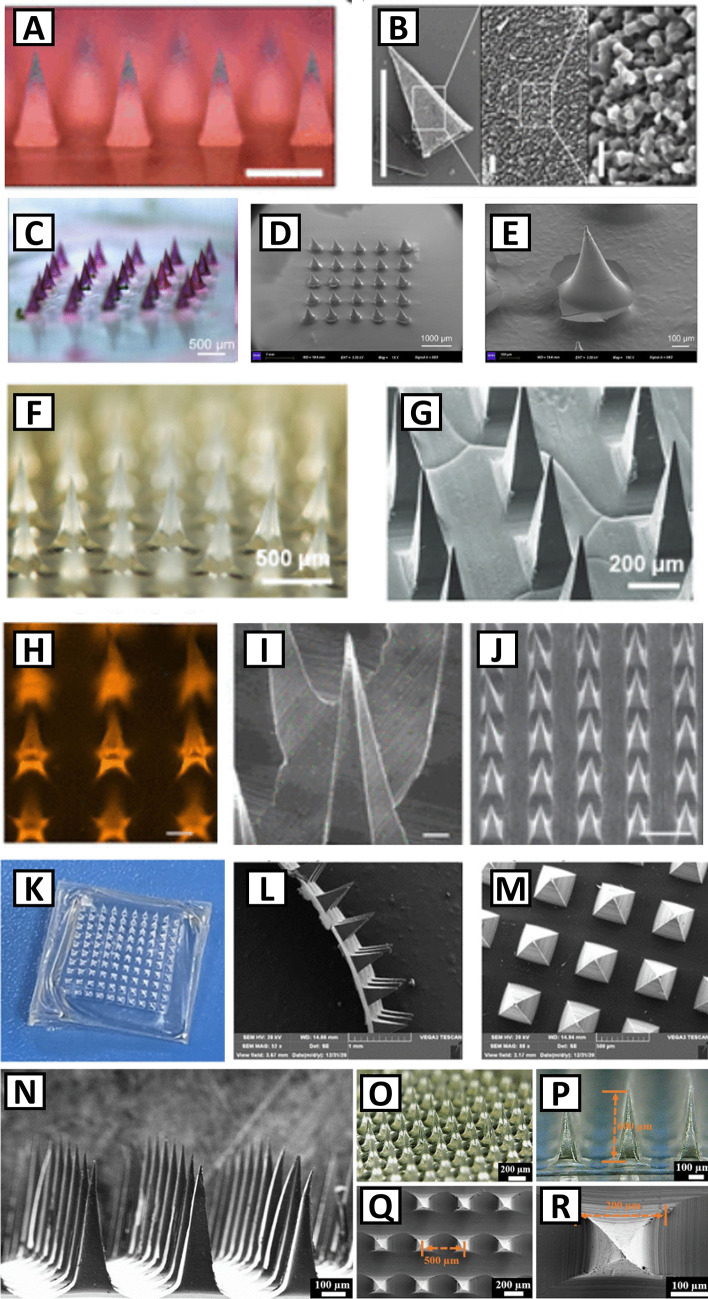
Silk-PAA composite microneedles: optical image (**A**) and SEM images (**B**) (scale bar: 500 µm) (adapted from ref. 47); gelatin-g-PNIPAAm MNs mounted onto PLA solid MNs coated with PVA: optical image (**C**) and SEM images (**D**, **E**) (adapted from ref.48); PNIPAAm-based MNs; optical image (**F**) and SEM image (**G**) (adapted from ref. 49); HAMA-based MNs: fluorescence microscopy image (scale bar: 250 µm) (**H**) and SEM image (scale bar: 50 µm) (**I**, **J**) (adapted from ref. 42); chitosan-based MNs: optical photos (**K**) and SEM images (**L**, **M**) (adapted from ref. 46); GelMA-based MNs: SEM image (**N**), bright field microscopy images (**O**, **P**), SEM images top view (**Q**, **R**). Adapted from ref. [45]

Shown in Fig. 2 are some examples of the size and shape of HFMs based on HAMA, chitosan, GelMA, PNIPAAm, and PAA-based HFMs [42, 45–49]. HAMA-based HFMs have been reported to exert a maximum force for skin penetration of 0.6 N with 100% swelling ratio (SR) for MNs of 250 µm base width and 850 µm height [42]. For GelMA-based MNs, maximum forces have been reported between 0.9 and 1.9 N, with SR of 50–70%, for MNs of 200 µm base and 600 µm height [45]. As an example of the properties of HFMs made of synthetic hydrogels, maximum forces for tissue penetration of 0.7 N have been reported for PNIPAAm, with tunable release and SRs depending on hydrogel composition [48, 49].

**Fig. 3:**
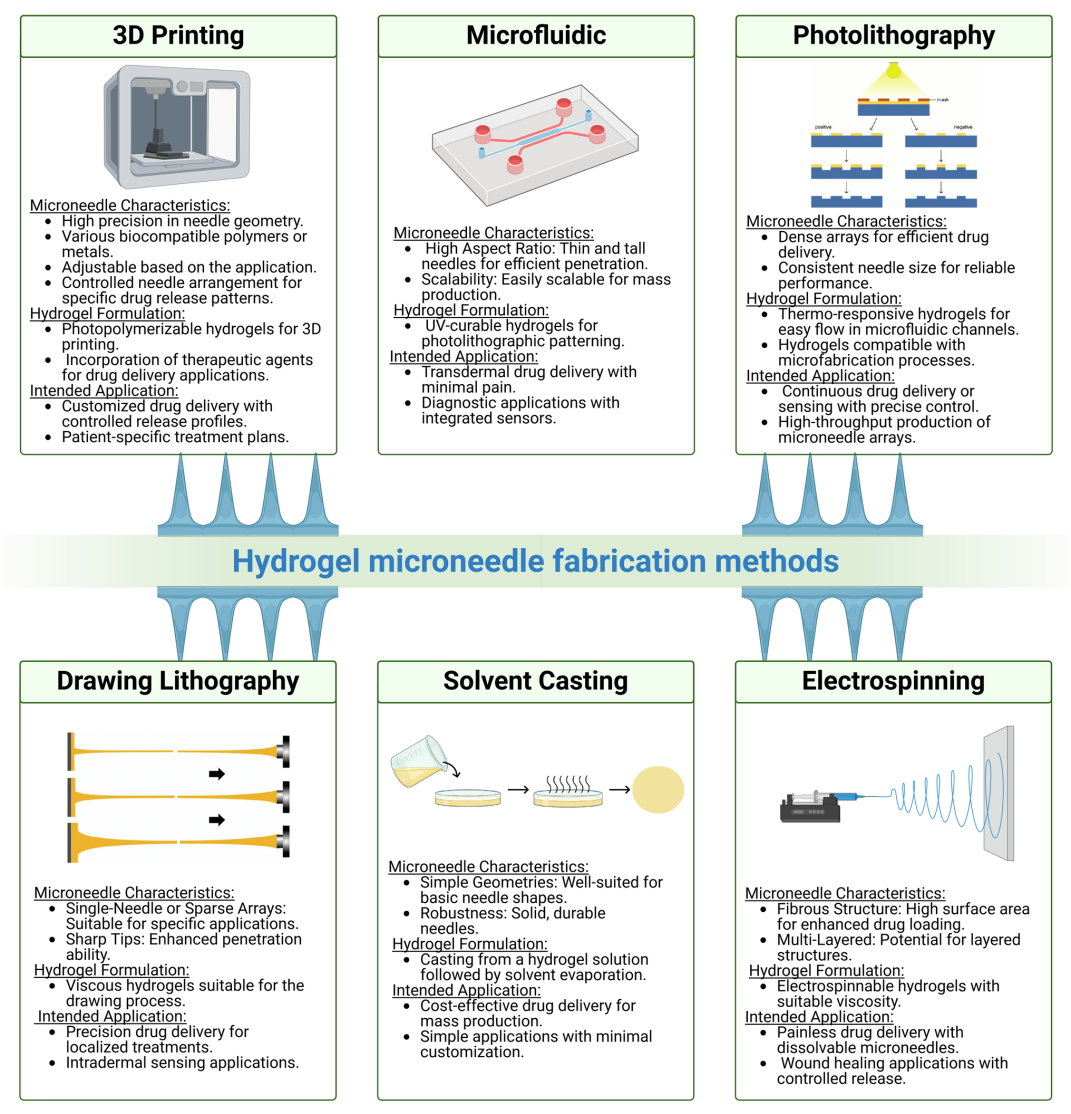
Schematic summary of the various fabrication methods used for the fabrication of HFMs for drug delivery. The choice of method depends on the specific hydrogel formulation, the desired MN characteristics, and the intended application. Researchers often select or combine methods based on the requirements of the targeted drug delivery system

Various methods exist for hydrogel microneedle fabrication, each with its own set of advantages and limitations (see Fig. 3) [27, 53]. The most established and widely accepted method for fabricating HFMs is micro-molding, which offers high reproducibility, convenient and scalable production, as well as cost-effectiveness. Other advantages of this method include low processing temperatures and an insignificant impact on drug activity. For micro-molding, hydrogels are casted into a micro-mold, which provides a precise control over microneedle dimensions and geometry. To ensure higher precision in the geometry and shape of the microneedle structure, laser ablation is often used to create the mold. Compared to other fabrication strategies, laser ablation permits an accurate control over the laser intensity and scanning speed, thereby becoming more versatile [27, 57]. However, micro-molding is limited to simple shapes and requires additional steps for removal of the MNs from the mold.

Photolitography is another useful technique, in which MN patterns are created on a substrate, followed by casting of a hydrogel into these patterns. This method allows a precise placement of the microneedle array, with well-defined microneedle shapes and easy scalability. However, complex and expensive equipment is required, and its use is limited to planar surfaces [58]. Recently, 3D printing has also emerged as a versatile tool for MN fabrication, involving layer-by-layer printing of the hydrogel to build MNs with controlled geometry. 3D printing offers customization and design flexibility, thereby opening the way to more complex structures. However, limiting factors such as printing speed, materials viscosity, and printability are to be considered, as well as the frequent need for post-processing steps [59]. Drawing lithography, in turn, achieves high precision by directly drawing MNs onto a hydrogel substrate, but it is restricted to specific formulations [60]. Solvent casting and particle leaching allow also for the creation of MNs by casting a hydrogel solution with dissolvable particles, allowing enhanced drug loading but with limited control over pore size [33]. Microfluidic fabrication utilizes controlled fluid flow in microchannels for precise microneedle geometry, but faces challenges in scale-up for mass production [61]. Finally, electrospinning produces nanofiber-based microneedles with increased drug loading, but lacks precise control over needle dimensions [62].

## Hydrogels and nanoparticles—smart platforms for nanotheranostics

Hydrogels and NPs, whether used separately or in combination, offer versatile platforms for nanotheranostics. The hydrogel provides a smart platform for drug delivery with controlled and targeted release of the therapeutic agent, additionally enabling combinatory therapies to address complex diseases, as well as incorporating contrast imaging agents for a theranostic approach [63, 64]. On the other hand, NPs made of polymers, dendrimers, liposomes, metals, and other inorganic materials are often used in nanotheranostics due to their potential therapeutic payload together with the enhanced permeability and retention (EPR) effect [26]. The EPR effect drives NPs to accumulate preferentially in tumors, making them suitable for cancer therapy [65, 66]. Theranostic NPs enable simultaneous imaging and treatment, providing real-time feedback on treatment efficacy and guiding therapeutic decisions.

NPs have excelled as drug delivery carriers by encapsulating small molecules, peptides, proteins, and nucleic acids, being more versatile and carrying higher payloads than conventional drug delivery systems. In cancer therapy, NPs may show a higher selectivity through either passive or active targeting mechanisms, which allow them to overcome some of the inherent limitations of conventional drugs by improving drug localization, enhancing drug solubility, and reducing toxic side effects through controlled release at the target site [67, 68]. Encapsulation of drug-loaded NPs within a hydrogel matrix has been used to prevent rapid drug release, both locally and systemically. NPs can in turn enable selective release by inducing a response in the hydrogel matrix under external stimuli. As an example, plasmonic metal NPs can generate heat in response to near-infrared (NIR) light irradiation, in turn inducing changes in the surrounding hydrogel that enhance drug release [69]. In this manner, NPs and hydrogel can improve the overall performance of the system through synergistic effects, such as increased absorption through the tissue of drugs with low permeability, prevention of particle aggregation, and stimuli responsiveness, contributing to a more specific control of hydrogel drug release [70].

**Fig. 4:**
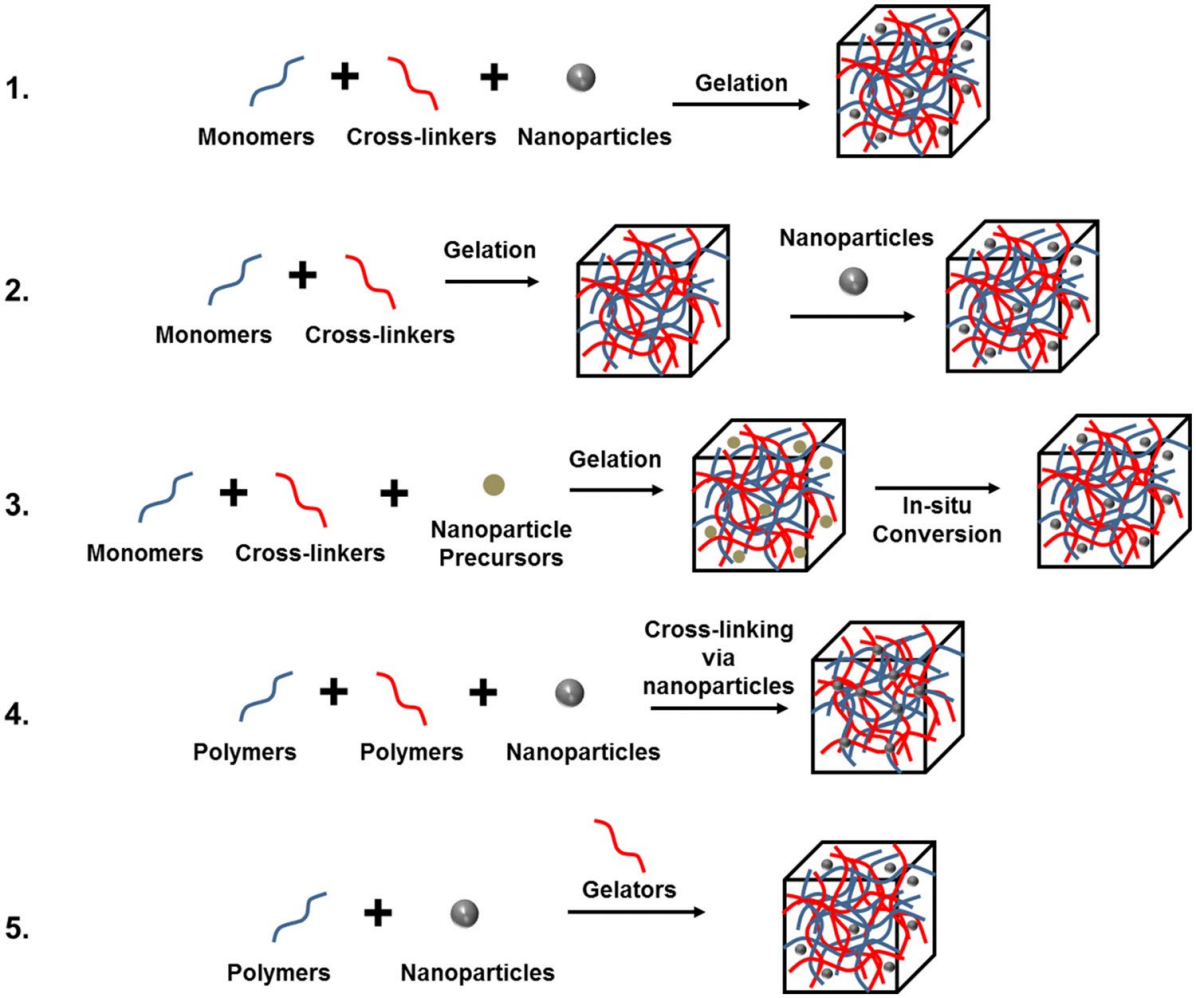
Representation of the main approaches used to obtain hydrogel‐nanoparticle conjugates with uniform distribution: (1) hydrogel formation in a nanoparticle suspension, (2) physically embedding nanoparticles in a hydrogel matrix after gelation, (3) reactive nanoparticle formation within a preformed gel, (4) crosslinking using nanoparticles to form hydrogels, (5) gel formation using nanoparticles, polymers, and distinct gelator molecules. Adapted from ref. [68]

The uniform distribution of NPs within a hydrogel matrix is crucial for applications such as drug delivery, tissue engineering, and sensing, as it ensures consistent and predictable material properties and performance. Summarized in Fig. 4 are the main approaches that have been reported toward a uniform distribution of NPs in hydrogels [68].

Each of these methods has its own advantages and may be chosen based on specific application requirements together with the properties of the NPs and the hydrogel. Factors to be considered when selecting the appropriate method include the desired NP distribution, NP size and surface properties, compatibility with the hydrogel matrix, and the overall purpose of the composite material. The simplest method involves the use of a hydrogel-forming monomer solution together with a suspension of NPs. By means of this approach, one can guarantee an even NP distribution throughout the entire hydrogel matrix [23, 24] The addition of NPs may reinforce the mechanical stability of the starting hydrogel, stabilize the NPs themselves, and provide responsiveness toward external stimuli [71, 72]. In terms of nanocomposite HFMs, Table 3 shows the different platforms used in drug delivery, ranging from polymeric to metal/metal oxide NPs.

**Table 3:** Types of NPs used for nanocomposite HFMs and their applications in drug delivery

**Nanoparticles**	**Polymer for HFM**	**Application**	**Ref**
Polycaprolactone (PCL)	PAA	Enzyme-responsive NPs for wound healing	[73]
Polyethylene oxide (PEO)	Gelatin	Microneedle electrosprayed with nanoparticles for skin treatment	[74]
PNIPAAm	PLGA	Hydrogel swelling triggering biodegradable microneedles for transdermal delivery	[31]
PLGA	PMVE/MA	Microneedles with nanoparticles for antitumor and antiviral responses	[75]
Chitosan	PVA	Microneedle-based photothermal therapy to initiate antitumor immunity and sensitize tumors	[76]
Lipid nanoparticles	PLGA	Microneedles for enhanced transcutaneous vaccine delivery	[77]
	PVP	Microneedles for synergistic chemo-immunotherapy	[52]
Gold nanorods	PVA	Light-triggered microneedles for superficial tumor treatment	[78]
	HA	Light-triggered microneedles for human epidermoid cancer treatment	[42]
	PLLA	Light-triggered microneedles for superficial tumor treatment	[79]
Metal oxide NPs	HA	Microneedles to treat tumor tissues by photodynamic therapy and chemotherapy	[80]

To achieve the desired performance, a better understanding of NP–matrix interactions and the mechanisms involved in the formation of these composite systems is needed. Special attention should be paid to the following: effective incorporation of the NP component to maximize therapeutic loading while maintaining the integrity of each component; tunable mechanical performance to match application-specific requirements; biocompatibility, which is essential in all biomedical applications but may be compromised during gelation or degradation; and long-term stability to minimize treatment frequency [81]. Achieving a uniform distribution of NPs in HFMs is thus a multidimensional challenge that requires careful consideration of hydrogel formulation, NP properties, and fabrication methods. Advanced imaging and spectroscopic techniques are valuable tools for characterizing and optimizing NP distribution within the hydrogel matrix, ultimately enhancing the performance of hydrogel microneedle drug delivery systems [81].

## Hydrogel microneedles in cancer therapy

Conventional cancer treatment procedures involve surgery, chemotherapy, and radiotherapy, all of which entail invasive methods, systemic toxicity, and drug resistance. The specific treatment differs for each type and stage of cancer, localization, and medical background of the patient. Surgery is the main approach to tackle cancer, but it is usually invasive, accompanied by risk of infection, hemorrhage, and complications from anesthesia [3]. Moreover, not all tumors are surgically accessible, and there is always a risk of recurrence if any cancerous cells are left behind. Chemotherapy is a broad and well-established therapeutic approach for cancer treatment that involves the use of drugs that target rapidly dividing cancer cells, to inhibit their growth or destroy them. It is considered a systemic therapy because the drugs circulate throughout the body, affecting cancer cells both at the primary tumor site and in other parts of the body where cancer may have spread (metastasized) [82]. However, chemotherapy often leads to toxic side effects and relapsing of cancer due to drug resistance development, ultimately resulting in a reduced patient quality of life [82]. Consequently, relying solely on a single-agent therapy does not consistently yield a favorable outcome for the cancer patient, and may result in undesirable side effects as well as an increased risk of cancer recurrence [82, 83].

**Fig. 5:**
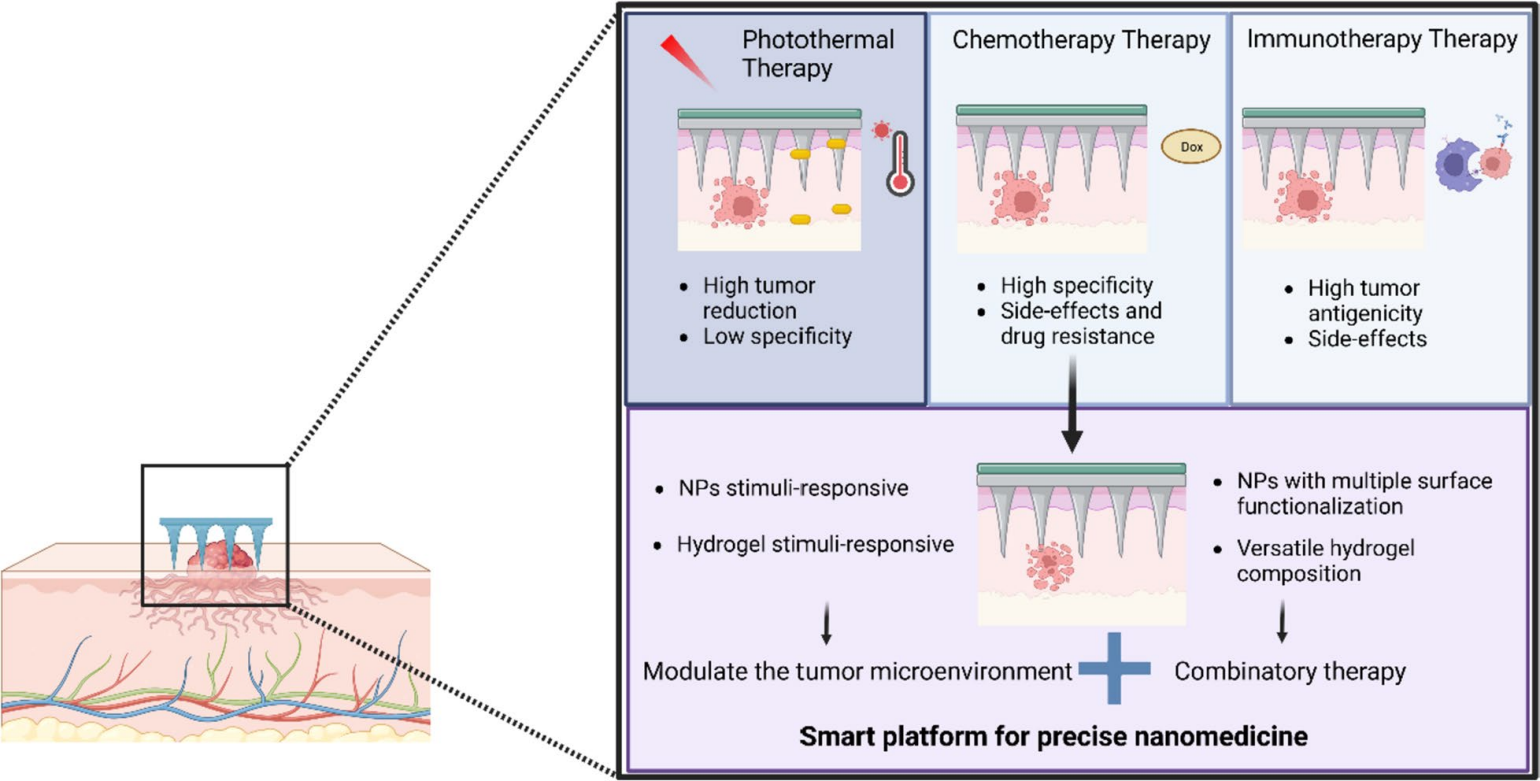
Representation of nanoparticle-loaded HFMs for cancer therapy. The table shows the main therapies where nanocomposite HFMs are used, detailing their benefits and drawbacks, as a tailored tool for personalized and precision medicine

An effective strategy is thus needed to address these challenges, with a better integration of therapies, so that synergistic benefits are effectively achieved, mitigating side effects and overcoming tumor cell resistance. A combined therapy entails three pivotal objectives that a single-agent therapy cannot achieve: (a) it provides a more specific treatment; (b) it reduces side effects, thereby increasing the quality of life of the patient; and (c) it lowers the required treatment dosage [84–86]. In this context, HFMs have emerged as a promising technology in cancer therapy, particularly in combination therapy approaches, including chemotherapy, immunotherapies, and other anticancer treatments (Fig. 5) [86]. Although HFMs have potential applications in various cancer therapies, the following aspects provide them with a specific relevance in melanoma treatment. HFMs are well suited for superficial lesions due to their ability to penetrate the skin at a suitable depth; they are minimally invasive, making them ideal for dermatology and skin cancer treatment [87]. By targeting the affected skin area with suitable therapeutic agents, systemic exposure is minimized and side effects are reduced. Therefore, HFMs can serve a dual purpose by not only delivering therapeutic agents but also enabling minimally invasive biopsy procedures for diagnostic sampling of melanoma lesions. Some hydrogel formulations used in MNs can be designed to respond to specific conditions, such as temperature changes associated with skin cancer, so that controlled drug release occurs selectively at the target site. As an example, HFMs made of hyaluronic acid for melanoma treatment have been reported to enhance the efficacy of immunotherapy, where the release of the antibody is triggered by pH-sensitive polymeric NPs. When the MNs enter the tumor acidic environment self-dissociation of NPs takes place, resulting in a more efficient process than intratumoral injection of free antibodies with the same dose [88].

For solid and superficial cancers, the combination of chemotherapy and photothermal therapy (PTT) can be used to enhance the therapeutic effect and improve treatment outcomes. Whereas chemotherapy acts at both the primary tumor and distant metastatic sites, PTT involves the use of light-absorbing sensitizers (molecules or plasmonic NPs) that have been previously released into or near the tumor [89]. When exposed to laser light of a specific wavelength, the sensitizers release part of the absorbed energy as heat, causing localized hyperthermia and selectively damage to cancer cells. The combination of chemotherapy and PTT is expected to have a synergistic effect, leading to increased cancer cell death. For example, hyperthermia induced by PTT can additionally enhance the uptake and effectiveness of chemotherapeutic drugs in the tumor microenvironment [90].

An appealing system for combined chemo-/photothermal therapy involves the use of HFMs loaded with both NIR-responsive NPs and anticancer drugs, thereby achieving a synergistic antitumoral effect [52]. Another example involves HFMs based on hyaluronic acid loaded with gold nanorods (AuNRs) and doxorubicin (DOX), again to combine the photothermal effect with the anticancer therapeutics, revealing remarkable antitumoral efficacy without any recurrence, after one single treatment [43]. Synergistic combinatory therapy has also been achieved using PCL MNs containing photosensitive molecules and DOX as the chemotherapeutic drug. The support array patch was made of a dissolvable PVA/PVP mixture, so that the MNs could be inserted in the target tissue with no need for surgical procedures, thereby allowing a uniform delivery of both heat and drugs to the tumor, which was eradicated within 1 week after a single administration with MNs [78].

## Challenges and future perspectives

Although significant progress has been made in the development of HFMs with embedded NPs, the synthesis and in vivo application of this particular type of nanocomposites are still facing multiple challenges. Of note is the clearance of the structure upon complete release of the encapsulated therapeutic agents. Despite the high biocompatibility of the individual components, their prolonged retention poses a risk of adverse effects, such as foreign body responses. This issue may be addressed by utilizing more sophisticated strategies for controlled degradation, potentially involving simultaneous control over polymer cleavage rates, variation of crosslinkers, and the use of homogenous polymer networks. Other challenges include achieving gelation at an optimal time, prior to implantation or in situ. Premature gelation increases the risk of low or uneven incorporation of NPs within the structure, in turn leading to suboptimal delivery of therapeutic payloads. Conversely, delayed gelation poses risks of delivery to undesired areas, rather than specific targeted delivery [34, 40]. Employing smart polymer systems that quickly form a gel in response to a specific stimulus may be a viable approach for addressing this issue. With NP-loaded HFMs, it is also essential to consider the complexity of the biological environment. Applications such as immune modulation and tissue engineering rely on the ability of the platform to interact with individual cells and the microenvironment, both adjacent to and inside the structure itself. As such, platforms designed for these purposes must incorporate components that can correctly interface with the body. Therefore, improving the interface between biomedical devices, such as HFMs and the human body, is crucial for enhancing device performance, safety, and patient comfort. Some strategies to be taken into consideration include surface modification of the needles to enhance biocompatibility by improving tissue interactions and minimizing immune reactions [91].

Needle design and material properties are also crucial to further minimize tissue damage during insertion. Moreover, sterilization and packaging are also important to avoid contamination and biodegradability, reducing the risk of long-term implantation or foreign body responses. Overall, assembling different NPs and hydrogel platforms into superstructures such as HFMs represents a promising methodology for addressing and overcoming the limitations that traditional therapeutics currently have. With continued development and improved understanding, we anticipate that superstructure platforms will become increasingly popular [92]. Besides improving the compatibility of the material with the body, the integration of various types of NPs can potentially impart novel functionalities to hybrid HFMs. The resulting nanocomposite HFMs can serve to monitor tissues or targeted tumor parameters, such as temperature and pH. For instance, the incorporation of photoluminescent rare earth–based nanothermometers or pH-sensing probes would enable continuous monitoring of the therapeutic treatment. Furthermore, combining different NPs within a single system holds promise for multimodal sensing and imaging capabilities. On the other hand, by introducing piezoelectric particles, HFMs might serve to modulate the mechanical properties of the tumor microenvironment, potentially affecting the mechanobiology of cancer cells and modifying their behavior [93, 94].

## Conclusions

The incorporation of nanomaterials within HFMs represents an attractive approach to tailoring the mechanical properties of hydrogels and/or providing the overall system with responsiveness to mechanical, thermal, magnetic, or electric stimuli. The versatility of both hydrogel and NP composition enables the development of a smart toolbox of HFMs, with the advantages of being minimally invasive and highly biocompatible. Further studies must be carried out to better understand the interactions, at different length scales, between the polymeric chains of hydrogels and the embedded NPs. In the case of drug delivery systems, such studies should include the interactions between the NPs interior and the drug(s) loaded into them. This knowledge will aid in predicting and modulating the mechanical and functional behavior of the composite. Grasping the complex relationship between structure (from nano to macro scale) and resultant properties is foundational. Such an understanding will empower researchers to custom design HFMs tailored for specific applications, be it drug delivery, tissue engineering, or sensing. As research progresses, HFMs are ready to redefine therapeutic interventions, offering solutions that are both efficient and patient-friendly.
